# Hippocampal CA1 activity correlated with the distance to the goal and navigation performance

**DOI:** 10.1002/hipo.22813

**Published:** 2017-12-14

**Authors:** Hugo J. Spiers, H. Freyja Olafsdottir, Colin Lever

**Affiliations:** ^1^ Division of Psychology and Language Sciences, Department of Experimental Psychology, UCL Institute of Behavioural Neuroscience University College London London UK; ^2^ Division of Biosciences, Department of Cell & Developmental Biology University College London UK; ^3^ Department of Psychology University of Durham Durham UK

**Keywords:** goal‐directed behavior, navigation, place cell, spatial memory

## Abstract

Coding the distance to a future goal is an important function of a neural system supporting navigation. While some evidence indicates the hippocampus increases activity with proximity to the goal, others have found activity to decrease with proximity. To explore goal distance coding in the hippocampus we recorded from CA1 hippocampal place cells in rats as they navigated to learned goals in an event arena with a win‐stay lose‐shift rule. CA1 activity was positively correlated with the distance ‐ decreasing with proximity to the goal. The stronger the correlation between distance to the goal and CA1 activity, the more successful navigation was in a given task session. Acceleration, but not speed, was also correlated with the distance to the goal. However, the relationship between CA1 activity and navigation performance was independent of variation in acceleration and variation in speed. These results help clarify the situations in which CA1 activity encodes navigationally relevant information and the extent to which it relates to behavior.

## 1 INTRODUCTION

The hippocampus is thought to serve navigation by creating a cognitive map of the environment which can be used to plan trajectories to future goal locations (Hartley, Lever, Burgess, & O'Keefe, 2014; O'Keefe & Nadel, 1978; Spiers & Barry, 2015). The spatially localized firing of hippocampal place cells in regions CA1 and CA3 of the rodent hippocampus has provided compelling evidence for the role of the hippocampus in forming a cognitive map of the environment (O'Keefe & Nadel, 1978). However, much less certain is how the hippocampus contributes to processing future goals or representations of the spatial relationship to goal locations (Poucet et al., 2004; Spiers & Barry, 2015).

A number of studies have found that dorsal CA1 place cell activity increases with proximity to goal locations (Dupret, O'Neill, Pleydell‐Bouverie, & Csicsvari, 2010; Eichenbaum, Kuperstein, Fagan, & Nagode, 1987; Fyhn, Molden, Hollup, Moser, & Moser, 2002; Hollup, Molden, Donnett, Moser, & Moser, 2001; Hok et al., 2007; Kobayashi, Tran, Nishijo, Ono, & Matsumoto, 2003). It is thought such proximity coding may be evidence for the read‐out of a stored cognitive map of the environment (Epstein, Patai, Julian, & Spiers, 2017; Spiers & Barry, 2015). Place cells in rats have been shown to express an extra firing field at an unmarked goal location in an open arena when navigating based on memory, but not when the goal location was marked by a visual cue (Hok et al., 2007). Similarly, when rats learn a set of new goal locations in an open arena some CA1 place fields showed a shift to being located more proximal to the goal, but not when goals were marked by visual cues (Dupret et al., 2010). Recent evidence indicates that CA1 neurons in bats navigating to a goal show activity tuned to the distance to the goal (de Cothi & Spiers, 2017; Sarel, Finkelstein, Las, & Ulanovsky, 2017), with more neurons tuned to distances proximal to the goal. These rodent and bat studies have tended to use open field environments and indicate that global population activity in CA1 would tend to increase with proximity to the goal, conforming to models in which navigation is guided via a gradient accent of activity to the goal (Bilkey & Clearwater, 2005; Burgess & O'Keefe, 1996; Trullier & Meyer, 2000).

However, not all studies have found activity increasing near goal locations. Several studies have found that place cell population activity decreases with proximity to the goal during navigation due to place fields being more numerous in the path at the beginning of the journey (Ainge, Tamosiunaite, Wörgötter, & Dudchenko,, 2007, 2012; Grieves, Wood, & Dudchenko, 2016). Such studies have used track‐based environments, requiring rats to make sequential decisions to navigate to a set of different goals. Recent work has also shown that when rats initiate a trajectory on a circular track the distance to the future goal is expressed in forward sweeps of the ensemble during theta state (Wikenheiser & Redish, 2015), consistent with the ensemble activity being greater when the rat is further from the goal. Evidence of greater hippocampal activity when farther from the goal is consistent with models in which the rat stimulates the future trajectory ahead to help plan the path (Bush, Barry, Manson, & Burgess, 2015; Erdem & Hasselmo, 2005; Penny et al., 2013). This is because each step along a simulated path will require activation of cells representing that fragment of space, and thus the longer the path the more cells that will need to be activated to represent the space ahead of the rat.

Mirroring the rodent and bat studies, human functional magnetic resonance imaging (fMRI) studies have also provided conflicting results when exploring goal distance encoding. Some studies have found hippocampal activity to increase with proximity to the goal (Balaguer, Spiers, Hassabis, & Summerfield, 2016; Howard et al., 2014; Patai et al., 2017; Sherrill et al., 2013; Viard, Doeller, Hartley, Bird, & Burgess, 2011), other have reported a decrease (Chrastil, Sherrill, Hasselmo, & Stern, 2015; Howard et al., 2014; Spiers & Maguire, 2007). It is currently unclear across methods and species whether activity in the hippocampus will tend to increase or decrease with proximity to a remembered goal. Moreover, little research has explored whether the correlation with distance is related to performance. If goal distance coding is important for navigation then navigational accuracy should co‐vary with the strength of the relationship between CA1 activity and the distance to the goal. Consistent with this, several neuroimaging studies exploring spatial navigation have found a correlation between the amount of evoked hippocampal activity during navigation and performance accuracy (Hartley, Maguire, Spiers, & Burgess, 2003; Maguire et al., 1998; Rauchs et al., 2008; Sherrill et al., 2013; Xu, Evensmoen, Lehn, Pintzka, & Håberg, 2010).

Here, we explored whether activity in the rodent CA1 hippocampus increased or decreased with proximity to the goal during navigation in an open‐field “event arena,” and whether CA1 activity correlated with the distance to the goal might reflect a code supporting navigational performance. We used a task where reward shifted between two goal locations within a given session.

## 2 METHODS

### 2.1 Subjects

Three male Lister Hooded rats (300–450 g in weight) were housed individually [11:11 light:dark, with 1 hr (×2) simulated dawn/dusk] on a food‐restricted diet sufficient to maintain 90% of free‐feeding weight, with ad libitum access to water. All procedures were licensed by the UK Home Office subject to the restrictions and provisions contained in the Animals (Scientific Procedures) Act 1986.

### 2.2 Apparatus

**Figure 1:**
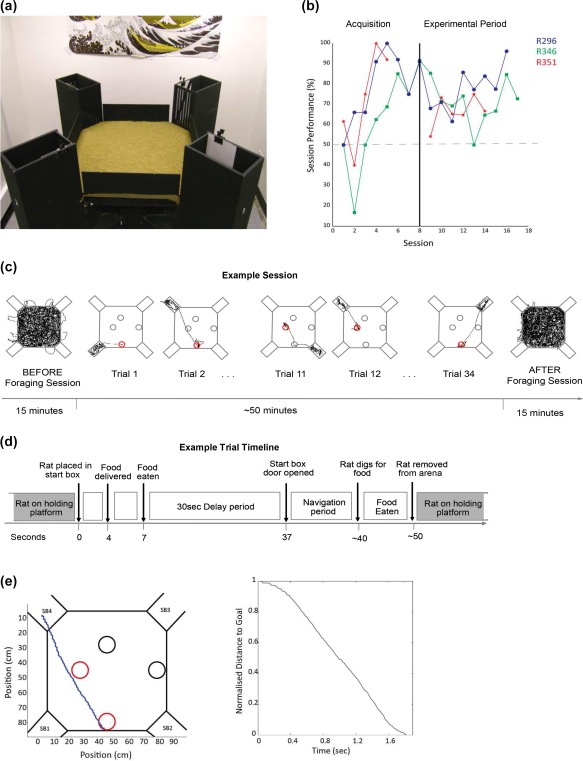
Apparatus and experimental task. (a) Photograph of the experimental apparatus. The arena floor was covered with sand, obscuring the goal wells. Each corner of the arena has a start‐box with a unique visual cue clipped inside on the rightwards wall (based on facing into the arena from the start box). (b) Learning curves for the three rats with % correct trials per session plotted against session number. The first eight sessions represented acquisition sessions where animals only had to run to one goal well (either South or West) in a session. Due to rapid acquisition by R351, the acquisition period was shorter (five sessions). Following acquisition (> 80% performance in two of three sessions), the animals carried out the experimental protocol where they either ran to the South or West goal well. The gray dashed line represents chance performance and the black vertical line the separation between the training and experimental period. (c) Session timeline. Each session began with a 15‐min foraging session, followed by 14–40 goal‐trials. Images show the tracked position (black) of a rat in the arena. During the goal trials animals ran from one of four start boxes to either the south or west goal wells (marked in red), depending on the food they ate while in the start box. Following goal‐trials, the animals completed another 15‐min foraging session. Event arena walls are marked in gray. The rat's trajectory is marked in black. Circles indicate the position of goal‐wells. The red circle indicates the rewarded goal in each trial. (d) Trial timeline. A schematic of a trial time line. Before a trial began, the rats sat on a holding platform. From there they were moved to one of four start boxes, in which they received food (chocolate or banana). It generally took the animals 3–4 s to locate the food and consume it. On food consumption, a 30 s delay period began, after which the start box was opened and the animal had to navigate to the goal‐well associated with the food it received in the start box (“Navigation period”). Once the animal had dug for food, the rat was removed from the arena and put back on the holding platform. Each trial lasted proximately 1 min. (e) Example of a trial trajectory (blue) and a plot of the distance to goal against time for this trial. SB = Start box. Trial start was defined as 250 ms prior to the animal crossing any of the entries into the arena (diagonal corners). A trial end was defined as 500 ms after entry into goal zone [Color figure can be viewed at http://wileyonlinelibrary.com]

The experiment was carried out in an “event arena” environment made of wood painted with matte black paint containing a sand filled floor (see Figure 1a). The arena was surrounded by a set of fixed distal cues. The 80 cm × 80 cm platform in the event arena was at a height of 100 cm in the room and covered with sand approximately 1 cm deep. The edges of the platform had 14 cm high walls such that the distal cues were clearly visible to the rats. The arena floor had 4 locations in which sand‐filled tubs (“goal‐wells,” diameter 11 cm, 6 cm deep) were placed into holes cut in the arena floor such that the top of the open tub was flush with the floor of the arena. Two steps were taken to reduce the possibility that rats were choosing the well using olfactory cues. Each had a wire mesh grid half way down, below the grid the wells were filled with sand and a ground up mix of the two food types used in the experiment. In addition, the sand in the wells was mixed with an equal amount of the two foods. Above the grill the tub was filled with sand to the top. The sand in the wells was at a level with the sand in the arena floor, such that they were not visually identifiable (see Figure 1a). Four “start boxes” were located at each corner of the arena. These were 30 cm high, 10 cm wide, and 30 cm in length. Each one contained a distinct visual cue (white card, white card with a diagonal, black card, or white card with a large back circle. Each had a door with a grill cut with 1 cm wide bars, so that the rat could view the arena when in the start box. Between every session, sand was moved thoroughly around the apparatus and mixed with new sand and the wooden painted parts of the apparatus wiped down with water. A holding platform (10 cm × 10 cm, 1 m height) was located 1m east of the arena.

### 2.3 Surgery and electrodes

All rats were implanted at the start of the experiment with moveable microelectrodes. Four tetrodes were constructed from 4 interwound 25‐µm diameter platinum‐iridium wire (California Fine Wire). The tetrodes were held in a microdrive assembly (Axona Ltd, St Albans, UK) that allowed them to be lowered or raised with one full turn of the screw equal to an increment of 200 µm dorso‐ventrally. For 2 rats microdrives were implanted in both left and right hemispheres, for the other rat the implant was in the right hemisphere. The animals were premedicated with buprenorphine and anesthetized with isoflurane and oxygen (3 L/min) before being placed on a stereotaxic frame, with lambda and bregma in the horizontal plane. Microdrives were fixed to the skull with six 1.6‐mm jewelers' screws (Precision Technology Supplies Ltd) and dental cement. One of the screws was soldered to a ground wire to enable the animal to be electrically grounded. The electrodes were lowered into the neocortex above the dorsal CA1 region of the hippocampus. Once the electrodes were implanted, a metallic sleeve was pulled down over the remaining exposed wires. Postsurgery the animals were monitored periodically until they awoke. All animals were given at least 1 week to recover following the surgery and treated with the analgesic for 3 days.

### 2.4 Recording

Screening for place cells commenced 1 week after surgery, and took place in an area beyond the curtained environment in separate room to testing. Recording of single neuron activity was done using multichannel recording equipment (DacqUSB, Axona Ltd). The rats were connected to the recording device via lightweight wires and a socket attached to the microdrive plug. The potentials recorded on each of the 16 electrodes of the 4 tetrodes were passed through an AC‐coupled, unity gain operational amplifiers, mounted on the rat's head and fed to the recording system. The signal was amplified (∼20 000 times), bandpass filtered (300 Hz–7 kHz) and then collected and stored on a computer. Each of the 4 wires of one tetrode was recorded differentially with respect to a wire from one of the other tetrodes. A headstage with 1 infrared LED array was used to track the rat's location at a rate of 50 Hz. Video was recorded from a camera mounted above the middle of the arena. Once complex spikes were identified on the oscilloscope trace, the rats foraged on a 60 × 40 cm platform scattered with rice cooked with honey while screening for place‐specific unit activity was undertaken. If no place cell activity was present, the electrodes were lowered by around 50 µm. In total, the dataset consisted of 385 cells recorded from 15 sessions (5 sessions per rat, 26 ± 3 place cells per session—see Table 1).

**Table 1:** Session statistics

Rat	Session	Max cells	Performance (% correct)	# Trials	# Trials included	South trials included	West trials included
**296**	9	23	68	30	23	9	14
**296**	10	21	70.97	31	27	17	10
**296**	12	18	85.71	21	19	11	8
**296**	15	10	77.5	40	38	16	22
**296**	16	7	96.16	26	24	8	16
**346**	9	24	85.29	34	32	9	23
**346**	12	44	74.07	27	25	10	15
**346**	15	43	66.67	39	33	10	23
**346**	16	45	84.62	27	27	10	17
**346**	17	42	72.73	19	19	5	14
**351**	10	22	73.33	30	16	7	9
**351**	11	25	65.22	23	16	7	9
**351**	12	23	64.71	34	25	13	12
**351**	13	19	75	16	13	2	11
**351**	14	19	66.67	36	27	14	13
**Mean**		**26**	**75.11**	**29**	**24**	**10**	**14**

### 2.5 Experimental procedure

The experiment was an adapted version of the task used by Tse et al. (2007). While Tse et al. (2007) trained animals to associate six different flavors with six different goals in an arena with 49 goal locations, we trained rats to associate 2 flavors with two goals in our arena with 4 goals. This was done to provide reliable navigation performance across trials and train rats in a shorter duration, aiding single unit recording. A variety of other adaptations to the Tse et al. (2007) protocol were used. For one rat pretraining started prior to surgery (R296), for the other two it occurred after surgery (R346, R351). Each rat was tested from start of the protocol to the end before the next rat began the experiment (order R296, R346, R351).

### 2.6 Pretraining and acquisition

On the first day, rats were allowed to explore the arena for 15 min freely. At the end of the session they were placed in each of the four start boxes for 30 s each. The next session was used to train the rats to dig in the sand for food. This involved placing a sand‐filled tub (11 cm diameter, 6 cm high) on top of the middle of the arena floor. A food not used in the recording sessions (“Curiously Cinnamon” squares, Nestle©) was placed in the tub. The rats were allowed to learn to dig from the tub for the food for 15 min. After this the tub was removed and the food was placed protruding from the sand in the four goal‐wells. The rat was allowed to travel between the four locations to dig for the food, with the food being submerged in subsequent visits, such that the rat learned to visit each of the goal‐wells. Rats were habituated to the food used for training and the other food used in the experiment (chocolate pellets and banana flakes), by placing a small quantity of each (< 2 g) in their home cage. Chocolate pellets and banana flakes were chosen because rats (not used in this experiment) in test trials consumed similar amounts of these foods relative to other foods. Banana flakes were cut to a size matched in calorific content to the chocolate pellets. Prior to performing the task for single unit recording rats were initially trained to a high level of performance (> 80% correct) in sessions with a single goal, switching goal locations between sessions (Figure 1b). After that time point, all sessions involved two different goal locations being tested in the same session.

### 2.7 Experimental task

During a single session (Figure 1c), there were two goal locations, only one of which was correct in a given series of trials. A “goal trial” began with the rat being placed in the start box and a particular flavored food being delivered into the start box. After the rat consumed the food a 30‐s delay began, after which the experimenter opened the door of the start box (see Figure 1d). To obtain the reward, the rat had to proceed to the correct goal‐well and dig there to uncover more of the same food that was delivered into the start box. The other three goal‐wells were not baited in a given goal trial. Navigation to two different goal locations was tested during a session, typically with repetitions (e.g., South, then West, then South, then West, see Table 1 for trials‐in‐session statistics). A trial was scored “Correct” when the rat's first dig occurred at the goal‐well. Start box location was randomized with each set of four trials (e.g., 1,2,3,4; 3,1,4,2). Each session began randomly with the south goal or west goal being baited. The south goal was associated with chocolate and the west goal with banana, for all rats. After rats had successfully navigated from each of the start boxes to the baited goal‐well, that goal‐well was no longer baited and the other goal‐well was baited. In between trials the rat rested on the holding platform for approximately 15–30 s. Two 15‐min “baseline” foraging trials were given, one BEFORE the navigation task and one AFTER (see Figure 1c), during which rats searched for rice flavored with honey scattered throughout the arena. CA1 place cells were recorded during goal trials and during these baseline foraging trials.

### 2.8 Data inclusion

Only putative pyramidal cell activity was analyzed. Putative pyramidal cells were distinguished from putative interneurons based on waveform properties. Namely, cells with narrow waveforms (peak‐to‐trough duration < 0.4 ms) and high firing rates (mean firing rate > 5 Hz) were excluded. Cells that fired over more than 75% of the total area of the arena, or that did not reach a peak firing rate of 0.5 Hz in the foraging sessions, were excluded from further analysis Spiers et al., 2015. The remainder was considered putative place cells.

We only considered sessions where the animal performed above chance levels (50%, assessed using a binomial test). Here, session performance was defined as the number of times the animal ran to the correct goal well (either west or south) divided by the total number of trials in a session. Animal R351 only had five sessions where it performed above chance. To ensure each animal contributed equally to the analysis, we chose five sessions for the other two animals. The criteria we used to select these sessions were (a) the animal completed at least 20 trials, (b) the sessions chosen needed to represent a range of performance scores.

Only trials where the animal's trajectory concluded at one of the two goal wells (south and west) and where it reached its destination within 15 s were included in the analysis. Following this criterion, on average, 85% of all trials in a session were included in the final analysis. A total of 364 trials were included in the analysis. See Table 1 for information regarding number of cells, performance and trials for each session.

The main analysis is based on the goal trial data – when the animal is actively navigating to its goal (“Navigation period”). We did not analyze activity during the delay period in the start box. Although one might hypothesize that place cell activity during this period might relate to the animal's future trajectory (i.e., during sharp‐wave ripple activity [Buzsaki, Horvath, Urioste, Hetke, & Wise, 1992; Diba & Buzsáki, 2007; O'Keefe & Nadel, 1978]), our relatively low cell yield precluded such an analysis. Thus, we focused the analysis on the “Navigation period.” Here we examined the population activity rather than the distribution of firing fields. This was because rats took paths that were not always through the same space making it hard to estimate reliably the existence of a place field.

### 2.9 Data analysis

#### 2.9.1 Trial definition

Trials started 250 ms before the animal crossed the boundary of the arena from one of the four start boxes (see Figure 1e). We chose this definition for the start of a trial as the data showed the animals started accelerating while they were still in the start box, immediately preceding their entry into the arena. Moreover, we found 250 ms captured the initial acceleration while not including many time points when the animal was immobile. We tried using a longer time window (i.e., 500 ms), however, this resulted in the inclusion of slow movement samples. A trial ended 500 ms after the animal entered the goal zone of its destination goal well. The goal zone was a 20 cm × 20 cm square centered on the goal well (diameter of goal wells = 11 cm). 500 ms was chosen to limit the inclusion of samples where the animal was moving slowly, but also long enough to include samples when the animal had reached its destination. Position samples when the animal's speed was less than 3 cm/s were excluded. The average trial duration was 2.44 s (*SD* = 1.58 s). See Figure 1e for an example representative trial trajectory. To note, position estimates were smoothed with a boxcar kernel (400 ms long).

#### 2.9.2 Spatial binning

To analyze the relationship between firing rates and distance to goal, the position vector of a trial trajectory was first normalized so that 0 represented positions at the goal and 1 the start of the trajectory. The normalized position vector was then divided up into 20 equally sized distance bins (i.e., bin size = 0.05 normalized distance units). See Figure 3 for a demonstration of binning. The indices of the position samples in each distance bin were used to bin the rates, speed and acceleration samples. As bins were defined in terms of distance travelled to goal, the time the animal spent in each bin varied. The average time spent per bin was 116.32 ms (*SD* = 101.12 ms).

#### 2.9.3 Goal trial analysis

In the goal trial analysis we wanted to estimate population activity while an animal navigated to a goal well. Consequently, estimates of population activity in each goal trial included both cells active in that trial as well as cells that may have been silent in that trial but active in other trials. However, to ensure silent cells did not exert too much weight we excluded cells that fired less than 10 spikes in a given goal trial session (on average 29% of all cells). In practice, the results of the main analysis were very similar with or without cells firing less than 10 spikes (population rates x distance to goal correlation: *r* = 0.74, including inactive cells: *r* = 0.75). Moreover, any samples where the rat's velocity was less than 3 cm/s were not included in the analysis. We estimated instantaneous firing rate for each 20 ms time bin (i.e., based on 50 Hz position sampling). Rates were rebinned into 20 spatial bins, according to the procedure described above. Namely, the indices for the positions in each spatial bin were used to assign rate samples to different spatial bins. We then computed a population activity vector by taking the mean rate at each spatial bin. Finally, to ensure trials contributed equally, we divided the rate in each distance bin by the maximum firing rate in a goal trial. To assess the relationship between population rates and distance, population rate vectors were correlated with distance to goal using the Pearson correlation coefficient (*r*). A correlation was deemed significant if it had an associated probability below 0.05. For the main analysis, population vectors for all 364 trials were averaged to get one activity vector which was correlated with distance to goal. This analysis was then broken down to look at animal/session differences, and goal‐well differences (i.e., trials ending at west vs. south goal).

#### 2.9.4 Speed and acceleration

For each trial instantaneous speed and acceleration were estimated at every 20 ms time bin (based on smoothed position estimates). Acceleration was defined as the change in speed between consecutive speed samples. Similar to rate, speed and acceleration were rebinned into 20 spatial bins, and correlated with distance to goals using the Pearson correlation coefficient.

#### 2.9.5 Foraging trial analysis

To assess whether population rates during goal trials reflected the distribution of place fields in the recording environment or “out‐of‐field” place cell activity we analyzed activity rates during the foraging sessions. The data from the foraging sessions that preceded and followed the goal trials were combined and used to control for position in the analysis looking at the relationship between population rates and distance to goal. That is, if population rates during trial trajectories vary as a function of distance to goals, one might suggest that this could result from place cells clustering around the start boxes. This would then create an artificial positive correlation with goal distance. Ratemaps for all recorded cells were generated by binning positions and spikes into 2 cm spatial bins, smoothing spikes and position (i.e., dwell time) separately with a Gaussian kernel (σ = 2 cm). Although low firing cells were excluded from the goal trial analysis we included them in the foraging analysis since the majority of cells met the activity threshold criteria for the goal trial analysis and the main results of that analysis were very similar with them included (*r* = 0.75 vs. *r* = 0.74). Firing rate for each ratemap bin was obtained by dividing the smoothed spikes by the smoothed dwell time. Once ratemaps for every cell had been generated, they were averaged (for both before and after foraging sessions) to create one population ratemap per session. For each goal trial, the positions sampled were extracted from the population ratemap, for the session the goal trial belonged to, maintaining the order in which positions were sampled in the goal trial (i.e., “foraging trials”). This gave the *expected* population vector. The expected population rate vector from these foraging trials was rebinned into 20 distance bins, similar to the trial rates. We then estimated the relationship between distance to goal and the expected population rates, obtained from the foraging trials, using a Pearson correlation. If the correlation between distance and rates in the foraging trials is similar to the one we obtain for that of the goal trials this would indicate the relationship between the population rates and distance in the goal trials is confounded by place field distribution.

#### 2.9.6 Performance analysis

To examine whether the relationship between population rates and distance to goal is modulated by navigational performance, each session was divided into two session blocks based on which goal was baited in each trial. That is, all trials when the south goal was baited belonged to one session block and all trials when the west goal was baited belonged to another session block. Since a total of 15 sessions were included in this analysis, dividing sessions into session blocks resulted in 30 blocks. We then estimated performance in each session block by dividing the number of correct trials in a session block by the total number of trials in a block. Moreover, we estimated the correlation between population rates and distance to goal for each session block. To do this, we averaged the rebinned population rates for all trials in a session block to obtain a session block population vector and then correlated this with distance to goal, using a Pearson correlation, as before. We then correlated the obtained Pearson correlation coefficients for each session block against performance. We repeated this procedure to estimate the relationship between performance and speed‐by‐distance correlations and acceleration‐by‐distance correlations.

## 3 RESULTS

### 3.1 Behavioral observations

Once animals had demonstrated learning during the acquisition period (when only one goal well was baited in a session) they consistently performed above chance when they carried out the version of the task where either of the two goal wells could be baited in a trial during the navigation period. A total of 364 trials were included in this analysis. Only sessions where the animals performed above chance level were included (50% correct). When performance for the two goal wells – south and west, are analyzed separately (i.e., data divided into “session blocks”) we observed a slightly higher mean performance for the south goal well—85.35% (*SD* = 12.18), than the west goal well—73.26% (*SD* = 18.35) *− t*(28) = 1.95, *p* = 0.06. This pattern is consistent with boundary‐related place cell firing whereby proximity to a boundary provides greater precision in spatial memory due to the reduced uncertainty about the location (Hartley et al., 2000; Lever, Burton, Jeewajee, O'Keefe, & Burgess, 2009), with the south goal well being proximal to a wall and the west goal well being off‐set from a wall (see Figure 1). To ensure this difference did not confound our results, we reran our main analyses for south and west goal trials separately.

### 3.2 Population rates vary positively with distance to goal

**Figure 2:**
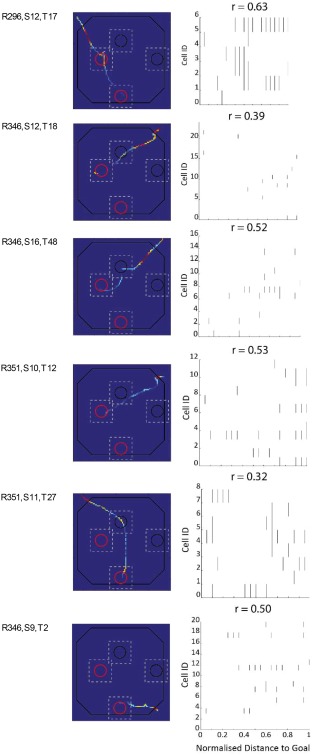
Trial examples of modulation of population firing rate by distance to goal. Examples from all three animals where population activity during a trial correlated positively with distance to goal. Left: Heatmaps of population activity in a trial. Hotter colors indicate higher population activity. Regions of the arena the animal did not traverse during a trial are colored in blue. Right: Raster plots of all cells recorded in a trial. Note spikes are plotted against normalized distance to goal (*x* axis). Plots clearly show more cells are active later. Title shows correlation between population rates and normalized distance to goal [Color figure can be viewed at http://wileyonlinelibrary.com]

**Figure 3:**
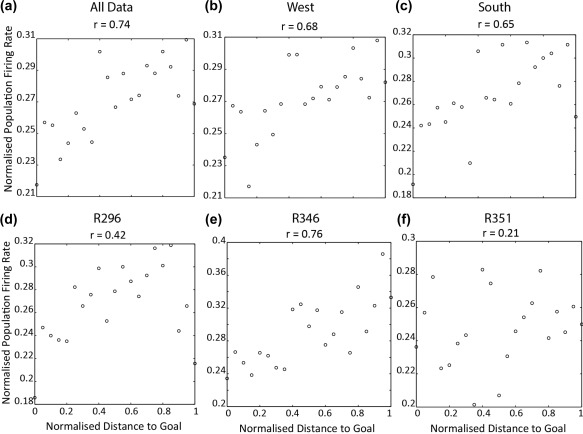
Distance‐to‐goal modulation on population rates. Scatterplot of population rates against normalized distance to goal. *x* axis shows distance to goal, and *y* axis the normalized population rates. The title of each plot shows the correlation between rates and distance. (a) Average population rates across goal distance for all data. (b) Average population rates across goal distance for all data where animal to west goal well, (c) same as (b) but for south goal well. (d–f) Population rates across distance to goal for R296, R346, and R351, respectively

To address the experimental hypothesis of whether population activity in the hippocampus is higher at navigational points where route planning is likely to occur, we assessed the relationship between population rates in a goal trial and distance to the destination goal well. Namely, distance to a goal was normalized and normalized population rate at each 0.05 distance unit estimated (see Methods). Figure 2 shows examples of population activity for several trials recorded, revealing a positive relationship between population rates and distance to goal. Moreover, we observed a significant, strongly positive, correlation between population rates and distance to goal when data from all trials was combined: *r* = 0.74, *p* = 1.40 × 10^−4^ (Figure 3a). Indicating rates were higher early in goal‐directed navigation, and that they dropped progressively as the animal moved closer to its goal. Importantly, a positive linear relationship between population rates and distance to goal was observed when trials going to south and west goals were analyzed separately (see Figure 3b,c) – south: *r* = 0.65, *p* = .0016, west: *r* = 0.68, *p* < .0001. Furthermore, when data for each animal was analyzed separately we obtained a positive correlation for all three animals, although only the correlation for animal R346 reached statistical significance (R296: *r* = 0.42, *p* = .057, R346: *r* = 0.76, *p* < .0001, R351 = 0.21, *p* = 0.37, see Figure 3d–f). In sum, population rates vary as a positive linear function of distance to goal, such that rates are higher at the start of a navigational trajectory than at the end. This is in agreement with our experimental hypothesis.

However, one might argue that this observed relationship between population rates and distance to goal may be a simple result of place field distribution; place fields may have been clustered around the start boxes resulting in an overall decreasing firing rate as a rat moves away from them and approaches its destination goal well. To explore this potential confound, we estimated the relationship between population rates and goal distance that would be predicted simply from locational ratemaps obtained from the foraging sessions.

### 3.3 Modulation of goal distance over population activity does not appear to be explained by place field distribution during random foraging

**Figure 4:**
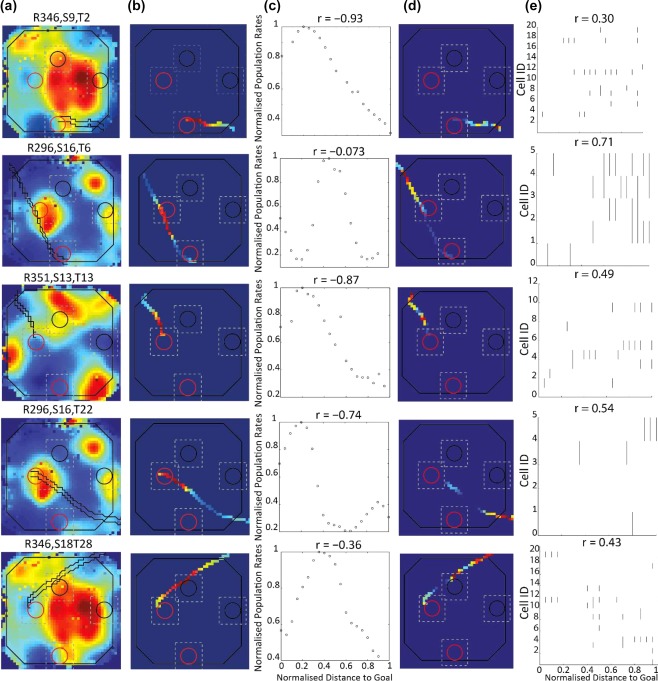
Goal distance modulation of population activity is not a result of place field distribution. Representative examples of population ratemaps (a), foraging trials (b), expected relationship between rates and goal distance (c), goal trial heatmaps (d) and observed relationship between rates and distance to goal (e). (a) Population ratemaps derived from foraging sessions, with an example goal trial trajectory shown in black. (b) Population ratemaps of a goal trial trajectory (shown in black in (a) based on rates in the foraging ratemap (“foraging trial”). (c) Expected population rates (derived from panel b) plotted against distance to goal. Title shows the correlation between rates and distance. *y* axis shows distance to goal and *y* axis normalized, expected population rates. (d) Heatmaps for goal trials. (e) Raster plots of goal trials shown in panel d), title shows correlation between population rates and distance to goal (*x* axis). Labels on top of ratemaps in panel a show rat, session and trial ID. A raster plot for foraging trials could not be constructed as each foraging trial is based on average activity over spatial bins, rather than instantaneous rate over a continuous run to a goal, as is the case for the goal trials [Color figure can be viewed at http://wileyonlinelibrary.com]

**Figure 5:**
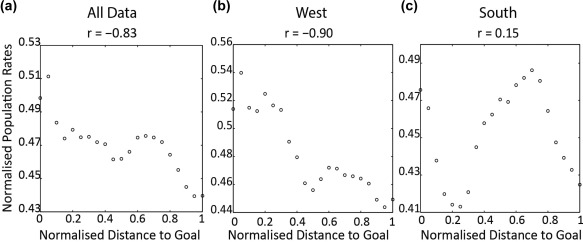
Relationship between distance to goal and population rates in foraging trials. Normalized population rates obtained from foraging trials plotted against distance to goal. (a) Rates for all foraging trials, (b) rates for all foraging trials concluding at the West Goal, (c) rates for all trials concluding at the South goal. *x* axis shows distance to goal, *y* axis normalized population rates, and title the correlation between the two axes

Before and after each goal trial session, animals completed a 15‐min foraging session. We used the ratemaps from these foraging sessions to estimate the expected relationship between population rates and distance to the goal (see Figure 4). Specifically, for every goal trial we extracted from the population ratemap the spatial bins sampled during the goal trial, maintaining the order in which the bins were visited and the dwell time in each bin (i.e., “foraging trials”). Similar to the goal trial analysis above, we rebinned the expected population vector into 20 distance bins and computed correlations between the rate vector for the foraging trials and distance to goal. We compared the correlation obtained from the foraging trials to those obtained from the goal trials. If the foraging trials’ correlations were positive like the correlations from the goal trials this would indicate that the relationship between goal distance and place cell activity in the goal trials merely reflected the distribution of place fields in the environment. Conversely, if the foraging trial correlations were different from those obtained in the goal trials this would imply the goal trial correlations reflect activity of place cells outside their main firing field. Figure 4 shows examples of these “foraging trials,” and the relationship between population rates and distance to a goal for each trial. These examples indicate that the observed relationship cannot be explained by place field distribution. The average correlation between distance to goal and predicted population rates was found to be strongly negative (Figure 5a)—*r* = −0.83, *p* < .0001, and was thus very different to the robust positive relationship observed for the goal trials (*r* = 0.76). Moreover, when analysis of activity estimated from foraging trial data were examined each goal separately, we obtained a strongly negative correlation for trials concluding at the West goal (*r* = −0.90, *p* < .001, Figure 5b), and a nonsignificant positive correlation for those ending at the South goal (*r* = 0.15, *p* = 0.51, Figure 5c). Although, the correlation between distance to goal and population rates for foraging trials ending at the South goal was positive, this correlation is considerably weaker, and nonsignificant, than the one we obtained for the goal trials (i.e., *r* = 0.66). The negative correlation obtained in foraging trials between population rates and distance to goal might imply place fields were clustered around goals, as found by previous work (Dupret et al., 2010). However, the weak correlation between population rates and distance to the south goal well is more consistent with place fields being clustered around the centre of the arena.

To conclude, we found a positive linear relationship between goal trial population rates and distance to goal is unlikely the result of place field distribution in the environment. Rather, the effect may reflect the activity of place cells when the animal is located outside their place field. Based on these results, one might speculate that the observed goal distance modulation is a result of task demands and thus that when goal‐distance rate modulation is stronger, the animal performs the task better. To address this question, we analyzed the relationship between navigation performance and goal‐distance rate modulation.

### 3.4 Navigational performance modulates the relationship between goal distance and population activity

**Figure 6:**
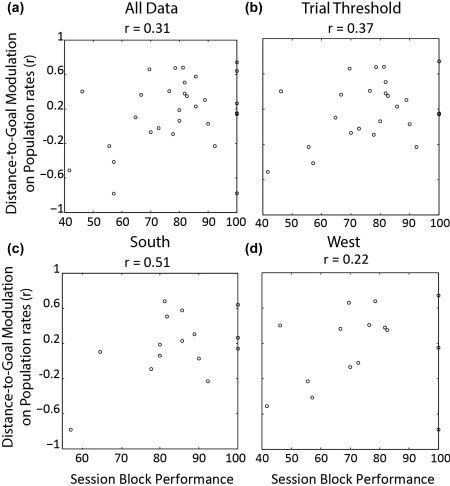
The influence of navigational performance over the relationship between population rates and distance to goal. (a) Correlation coefficients, derived from correlating distance to goal and population rates in each session block, plotted against session block performance. (b) Same as a) but only displaying data point from session blocks consisting of more than eight trials. (c and d) Same as (a) but where South and West session blocks are analyzed separately. The *x* axis shows session block performance, and *y* axis correlation coefficients. The title displays the observed correlation between the two axes

To assess whether the performance on the navigational task is systematically related to the observed positive relationship between population activity and distance to goal, we computed performance for each session block; each session was divided into two blocks based on which goal the animal ran to in a trial. Then the relationship between population rates and distance to goal was estimated, the obtained correlation coefficients were then correlated with the block performance. Overall, we observed a modest positive relationship between performance and distance modulation over population rates in session blocks (*r* = 0.31, *p* = .046, Figure 6a); implying the more accurately the animals performed in a session, the more positive the relationship between goal distance and population rates in that session. In other words, when an animal performed well, rates were highest early on in a trial. Moreover, when we analyzed the south and west session blocks separately we observed a similar trend (South: *r* = 0.51, *p* = .026; West: *r* = 0.22, *p* = 0.22, see Figure 6c,d). Visual inspection of a few outliers such as those at ceiling and near‐ceiling performance alerted us to consider the influence the number of trials in a block can have on performance measures. Namely, high performance scores may be more likely for blocks consisting of only a few trials. Consequently, we imposed an inclusion threshold on the session blocks, requiring each block to be composed of at least 2 trials from each start box (i.e., 8 trials in total). We then re‐estimated the correlation between block performance and the goal distance modulation over population activity. This increased the correlation *r* = 0.37, *p* = .036, see Figure 6b. In summary, not only do population rates in CA1 vary as a function of distance to goal, this relation seems to also be modulated by how well the animal performs. Although this correlation is modest, it is consistent across both the West and South session blocks.

### 3.5 The behavioral correlates (speed and acceleration) of population rates, distance to goal and navigation performance

Finally, we examined how running speed and acceleration vary as a function of distance to goal. It is known that place cell firing rates are positively correlated with running speed (Hirase, Czurkó, Csicsvari, & Buzsáki, 1999; McNaughton, Barnes, & O'Keefe, 1983). Firing patterns have also been shown to be modulated by acceleration (Gupta, Van Der Meer, Touretzky, & Redish, 2012) and the power of the theta rhythm has been found to correlate with acceleration (Long, Hinman, Chen, Escabi, & Chrobak, 2014). Thus, one might expect a correlation between place cell activity and acceleration. Perhaps at the start of a trial the animal's velocity is at its highest and then progressively drops as it approaches its destination goal. This would consequently lead to a positive relationship between cell activity rates and distance to goal, yet confounded by running speed. Moreover, it is conceivable that when the animal is performing well the positive relationship between running speed and distance to goal is even stronger; in other words, the correlation between velocity/acceleration and distance to goal may be modulated by performance. This would consequently also confound the reported performance correlations with the distance‐to‐goal modulated population rates. To address these possible confounds, we assessed the relationship between running speed and acceleration and distance to goal as well as the correlation between performance and the distance‐to‐goal modulation over speed/acceleration.

To assess whether the reported effects are a simple result of systematic variations in running speed/acceleration as an animal progresses through a navigational trial we performed a series of correlations. First, we estimated the relationship between population rates and running speed and acceleration. We observed a positive correlation for both running speed and acceleration (speed vs. rates: *r* = 0.51, *p =* .018, acceleration vs. rates: *r* = 0.67, *p* < .0001, Supporting Information Figure S1a,b). However, the relationship between distance to goal and speed was not nearly as strong, (*r* = 0.40, *p* = 0.07, see Supporting Information Figure S1c). Moreover, importantly, when we performed a partial correlation analysis between population rates and distance to goal while controlling for running speed, population rates still had a strongly positive and significant relationship with goal distance (*r* = 0.67, *p* = .0011). By contrast to speed, the correlation between acceleration and goal distance was found to be strongly linear and positive (*r* = 0.94, *p* < .0001, see Supporting Information Figure S1d), creating a potential confound in our analysis of this well‐learned task. Performing a partial correlation between activity rates and goal distance while controlling for the effect of acceleration did reduce the correlation between rates and goal distance such that it no longer reached statistical significance (*r* = .33, *p* = 0.16). To explore the effect of the potentially confounding relationship between acceleration and goal distance, we performed an alternative control analysis. We split the data in two: trials which showed a significant correlation between acceleration and distance to goal (*N* = 230, Pearson correlation, *p* < .05, resulting in *r* = 0.63, *SD* = 0.12) and those that did not (*N* = 134, *p* > 0.05, resulting in *r* = 0.29, *SD* = 0.14) and then repeated the main analysis, correlating population rates with distance to goal, separately for the two datasets. Importantly, we replicated our main results for *both* datasets (acceleration‐correlated trials: *r* = 0.58, *p* = .0056; acceleration‐uncorrelated trials: *r* = 0.70, *p* = .0004), see Supporting Information Figure S2. Thus, if anything, the relationship between rates and distance to goal was clearer in the acceleration‐uncorrelated trials. Furthermore, when we repeated these analyses while explicitly controlling for acceleration, the uncorrelated dataset maintained a significant correlation between rates and distance to goal (partial correlation, *r* = 0.56, *p* = .0087). In other words, in a dataset where acceleration and distance to goal could be teased apart, the main finding held: CA1 place cell activity strongly predicted distance to goal. As expected, the correlated dataset no longer showed a significant correlation between rates and goal distance once acceleration had been controlled for (*r* = 0.035, *p* = .88). In summary, we would argue that the strong relationship between acceleration and distance to goal in this task obscures but does not account for the relationship between CA1 population rates and goal distance.

Having explored the relationship between acceleration and distance to the goal we examined whether the relationship between activity and performance was confounded by speed and acceleration. We correlated session block performance with the correlation coefficients between speed/acceleration and distance to goal for each session block. For velocity we obtained a weak, positive relationship between performance and the extent to which goal distance modulated running speed in a session block, which did not reach statistical significance: *r* = 0.26, *p* = 0.083, see Supporting Information Figure S3. The relationship between acceleration and goal distance did not appear to be influenced by session performance: *r* = 0.095, *p* = 0.31, Supporting Information Figure S3. Therefore, it is unlikely that the reported relationship between session block performance and the modulatory effect of distance to goal on population rates is a direct confound of speed or acceleration. Moreover, in our performance analysis described above we reported stronger effects after removing blocks containing eight or fewer trials. Consequently, we applied the same criterion here and re‐estimated the correlation between session block performance and the relation between speed/acceleration and goal distance. Although this had the effect of increasing slightly the correlations, the increase was modest and the relationship did not reach statistical significance (speed: *r* = 0.31, *p* = .073, acceleration: *r* = 0.19, *p* = .19, see Supporting Information Figure S3). Finally, we examined the relationship between the firing rate distance modulation and session performance for the trials where acceleration was not significantly correlated with the distance. This revealed a significant correlation between the firing rate distance modulation and the session performance (*r* = 0.37, *p* = .036, Supporting Information Figure S4).

To conclude, the reported correlation between population rates and distance to goal does not seem to be a mere function of speed or acceleration. Running speed did not have a significant correlation with distance to goal, and only seemed to account for a small portion of the variance between activity rates and goal distance. Acceleration, conversely, did reveal a strongly positive relationship with goal distance, and across all trials did account for considerable variance between the place cell activity and distance to goal relation. However, in trials where acceleration was not correlated with the distance to the goal we observed significant simple and partial correlations between firing rate and distance. In addition, the session block correlation between goal distance and running speed/acceleration did not vary significantly with session block performance. Thus, the correlation between CA1 activity and navigation performance does not appear to arise purely from systematic variation in speed or acceleration.

## 4 DISCUSSION

We examined relationship between CA1 place cell activity and distance to the goal by record place cells in rats as they navigated from starting boxes to goals in an open‐field event arena. CA1 ensemble activity was found to be positively correlated with the distance to the goal, declining with proximity to the goal. This pattern could not be predicted from spatial firing patterns during the foraging sessions before and after the navigation task. Acceleration, but not speed, was also correlated with the distance to the goal and that variation in acceleration accounted for a significant amount of the relationship between CA1 activity and distance to the goal. Navigation performance in a session was more accurate when there was a stronger correlation between CA1 activity and distance to the goal and this was not found to be mediated by variation in acceleration or speed. These findings are consistent with CA1 supporting a cognitive map of the environment to guide goal‐directed navigation, as further discussed below.

Our finding that hippocampal activity is correlated with distance/proximity to a goal is consistent with similar observations in a number of studies (Ainge et al., 2007, 2012; Balaguer et al., 2016; Chrastil et al., 2015; Dupret et al., 2010; Eichenbaum et al., 1987; Fyhn et al., 2002; Grieves et al., 2016; Hollup et al., 2001; Hok et al., 2007; Howard et al., 2014; Kobayashi et al., 2003; Spiers & Maguire, 2007; Sherrill et al., 2013; Viard et al., 2011). This correlation pattern also agrees with the prediction from several computational models that population firing rate of some cell populations in the hippocampus should show a gradient of activity that varies with distance to the goal (Bilkey & Clearwater, 2005; Burgess & O'Keefe, 1996; Trullier & Meyer, 2000). In such models putative “goal cells” code the distance to the goal in their firing rate. While these models argued that navigation was supported by a gradient ascent process, where the animal navigates by maximizing activity, our data would rather be consistent with a gradient descent process where the goal is located by moving to minimize the activity of goal‐coding cells. More generally, a correlation between distance and hippocampal activity agrees with the view that the hippocampus serves navigation by coding information about the future path to the goal for navigation (e.g., Bush et al., 2015; Javadi et al., 2017; Pfeiffer & Foster, 2013; Poucet et al., 2004; Spiers & Barry, 2015).

The positive correlation between goal distance and activity we observed is consistent with the subset of studies that find a similar positive correlation between goal distance and hippocampal activity (Ainge et al., 2007, 2012; Chrastil et al., 2015; Grieves et al., 2016; Howard et al., 2014; Spiers & Maguire, 2007). Why do we and these other studies find a positive correlation between the distance to the goal and hippocampal activity, when others do not? One possible explanation may be differential task demands, for example, in terms of decision making and reward contingencies, including whether decision making is more taxing near the start or end of the journey. For instance, early‐in‐journey activity might reflect “getting there” processing, such as generating a vector to the goal, while later‐in‐journey activity might reflect “knowing where” processing, such as sensory identification of the goal from local cues. Whether “getting there” taxes hippocampal processing more than “knowing where” may depend on task set up. In our task and several others (Ainge et al., 2007, 2012; Brown et al 2010; Grieves et al., 2016; Howard et al., 2014; Spiers & Maguire, 2006; Wikenheiser & Redish, 2015) navigation required selecting a future path from a set of possible discrete paths. By contrast several studies that have reported activity increasing with proximity to the goal have reported this in tasks requiring localizing the goal in an open‐field in relation to distal cues and boundary information and where many different paths were used to reach the goal (e.g., Dupret et al., 2010; Hok et al., 2007; Sarel et al., 2017). While our task also required open‐field navigation, our rats tended to show, at least after training, relatively direct paths in navigating to the two goals from the four starting boxes, potentially indicating that the rats already knew where they were heading quite early on in the journey. Indeed, we designed the task with this possibility in mind. Accordingly, early‐in‐journey “getting there” processing may underlie the positive correlation between goal distance and firing rate, and the link between the strength of this correlation and navigation success. As for the studies that do not find either a positive or negative relationship between distance to the goal and hippocampal activity (e.g., van der Meer, Johnson, Schmitzer‐Torbert, & Redish, 2010), this lack of goal‐distance coding may be due to minimal demands to process self‐location or the goal location or little demand to suppress a set of trajectories in favor of the optimal path. It will be useful in future studies to vary the demands on self‐localization and use environments with terrain that can de‐correlate the distance along the path taken to the goal from the Euclidean distance to the goal (see Brunec, Javadi, Zisch, & Spiers, 2017; Howard et al., 2014). It will also be important to be able to assess the relative importance of normalized versus absolute distance to the goal. We employed two fixed goals and four starting points, which meant that, especially after training, many trials afforded only short start‐to‐goal journey distances. As with the Morris water maze task, once rats are well‐trained, while there is a reliable amount of datapoints for when the rat is at a short distance from the goal, there are relatively few trial segments when the rat is absolutely far from the goal. Using normalized distances meant we were able to gain statistical power from analyzing all the trials, and all the datapoints in a trial.

While the CA1 activity correlated with distance to the goal may relate to goal coding we also explored whether it might be more simply explained by other factors such as general place field distribution and changes in speed and acceleration. We did not find place cells were more likely to cluster near the start boxes during foraging. Moreover, the pattern of activity elicited during navigation periods did not match the predicted firing patterns based on foraging period place cell data. Thus, there appears to be a change in the way CA1 place cells respond when navigating compared with foraging in the same space. This is consistent with prior work showing “remapping” when rats switch from foraging to running a specific set of paths (Markus et al., 1995). Our data now provides evidence for how these changes occur when an animal shifts from foraging to goal directed navigation. Future research examining the relationship between place field distribution during navigation epochs with place field distributions during foraging will be useful to explore this topic.

The shift from foraging to navigation also contains a shift in how speed and acceleration may relate to firing rates. We explored whether the correlation between CA1 activity and distance to the goal might also relate to speed or acceleration and found acceleration, but not speed, was correlated with the distance to the goal. Further analysis revealed that a substantial amount of the variation in the correlation between CA1 firing rate and goal distance could potentially be explained by variations in acceleration. However, because firing rates were significantly correlated with distance to the goal (when controlling for acceleration) on the subset of trials where acceleration and distance were not correlated it appears that CA1 activity can reflect the distance to the goal to some independently of acceleration. It is also notable that a similar positive correlation between hippocampal activity and distance to the goal has been observed in rats and humans in contexts where acceleration was not a confounding variable (Ainge et al., 2007, 2012; Chrastil et al., 2015; Grieves et al., 2016; Howard et al., 2014; Sarel et al., 2017; Spiers & Maguire, 2007). Nonetheless, it is essential that future research dissociate the distance to the goal and acceleration to determine how CA1 activity correlates with information during goal directed navigation.

Our finding that the strength of the correlation between CA1 activity and distance was positively correlated with navigation session performance is consistent with several previous studies linking hippocampal activity to performance in humans (Hartley et al., 2003; Maguire et al., 1998; Rauchs et al., 2008; Sherrill et al., 2013; Xu et al., 2010) and rodents (Lenck‐Santini, Muller, Save, & Poucet, 2002; Ólafsdóttir, Barry, Saleem, Hassabis, & Spiers, 2015). This lends support to the view that the hippocampal code for goal distance is important in supporting navigational guidance (Spiers & Barry, 2015). However, we considered that this result might be mediated by variation in acceleration (or speed) occurring across the sessions. Our results suggest that was not the case, and that the link between CA1 activity and performance is not a simple function of speed or acceleration. Future research with more animals will be useful to determine if hippocampal activity‐to‐performance correlations help to explain both within‐subject trial performance, and across subject differences, as has been found with humans (Hartley et al., 2003; Patai et al., 2017). Moreover, given evidence of time coding cells in CA1 (Eichenbaum, 2014; Kraus, Robinson, White, Eichenbaum, & Hasselmo, 2013; MacDonald, Lepage, Eden, & Eichenbaum, 2011) it will be useful to separate the time taken to reach the goal from the distance travelled to determine whether the CA1 activity reflects distance, time or their conjunction in its activity code.

In conclusion, we find evidence to support the view that CA1 codes information about future goals during navigation and that the strength of this code predicts performance. Future experiments will be needed to systematically separate out the influence of other variables such as velocity, acceleration and path properties to understand how CA1 and other brain areas contribute to navigational guidance.

## Supporting information

Additional Supporting Information may be found online in the supporting information tab for this article.

Supporting Information
