# Supplementary material for: Hippocampal CA1 activity correlated with the distance to the goal and navigation performance
Source: Hippocampus. 2017 Dec 14;28(9):644–58. doi: 10.1002/hipo.22813 (PMC6282985; doi:10.1002/hipo.22813)
Supplement: Supplementary file 1 — Supporting Information [file HIPO-28-644-s001.docx]

**Supplementary Figures**


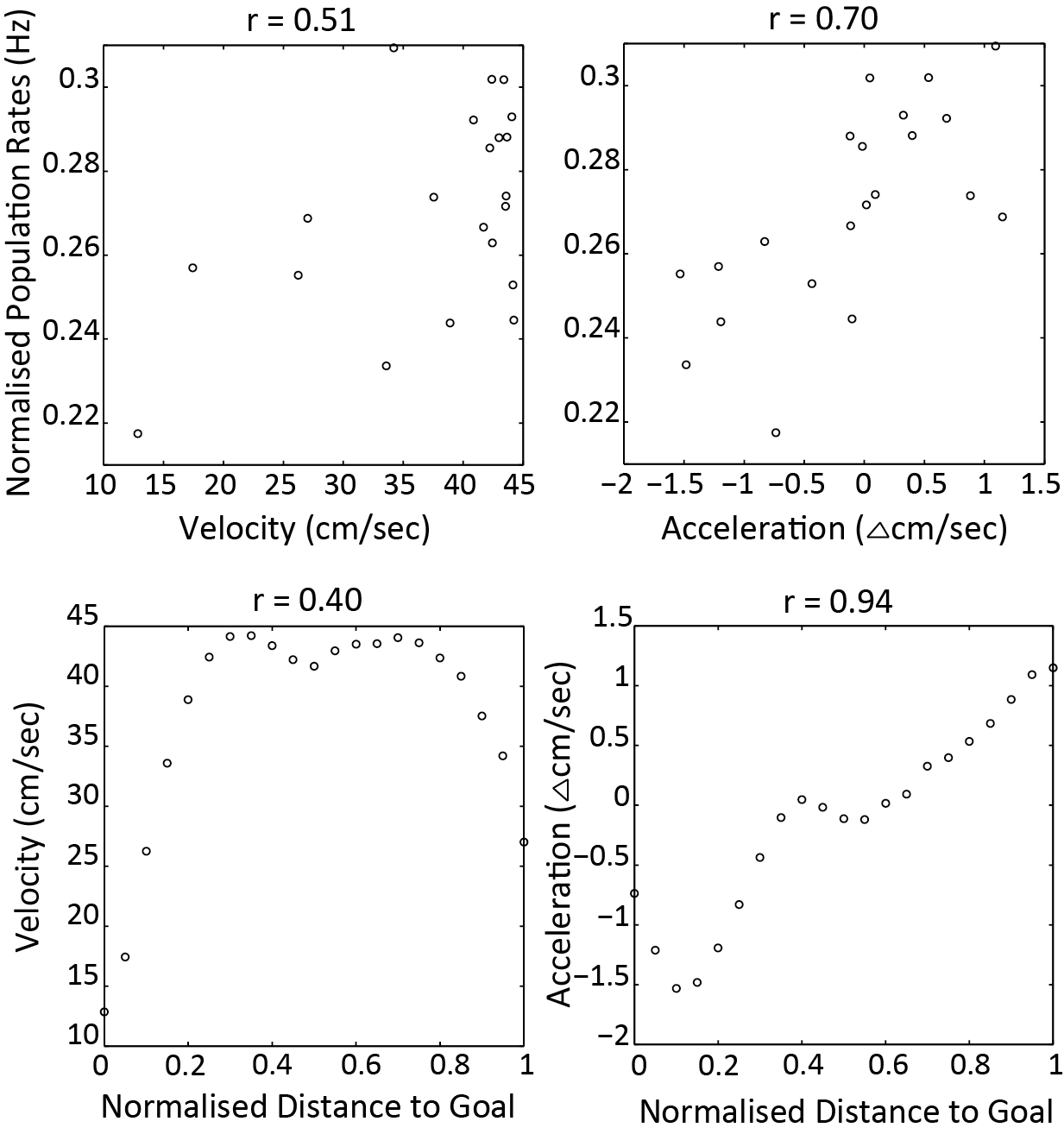


**Figure S1. The Relationship between Velocity/Acceleration and Population Activity and Distance to Goal.** **Top Left)** population rates plotted against velocity, **Top Right)** same as Top Left but where rates are plotted against acceleration. **Bottom Left)** Running speed, across all trials, plotted against distance to goal, revealing a non-linear relationship. **Bottom Right)** Same as Top Left but for acceleration. X-axis shows velocity and acceleration for top left and right panels, respectively and normalised distance to goal for bottom panels. Y-axis shows normalised population rates for top panels and the velocity/acceleration for bottom panels. The title demonstrates the estimated correlation between the two axes.


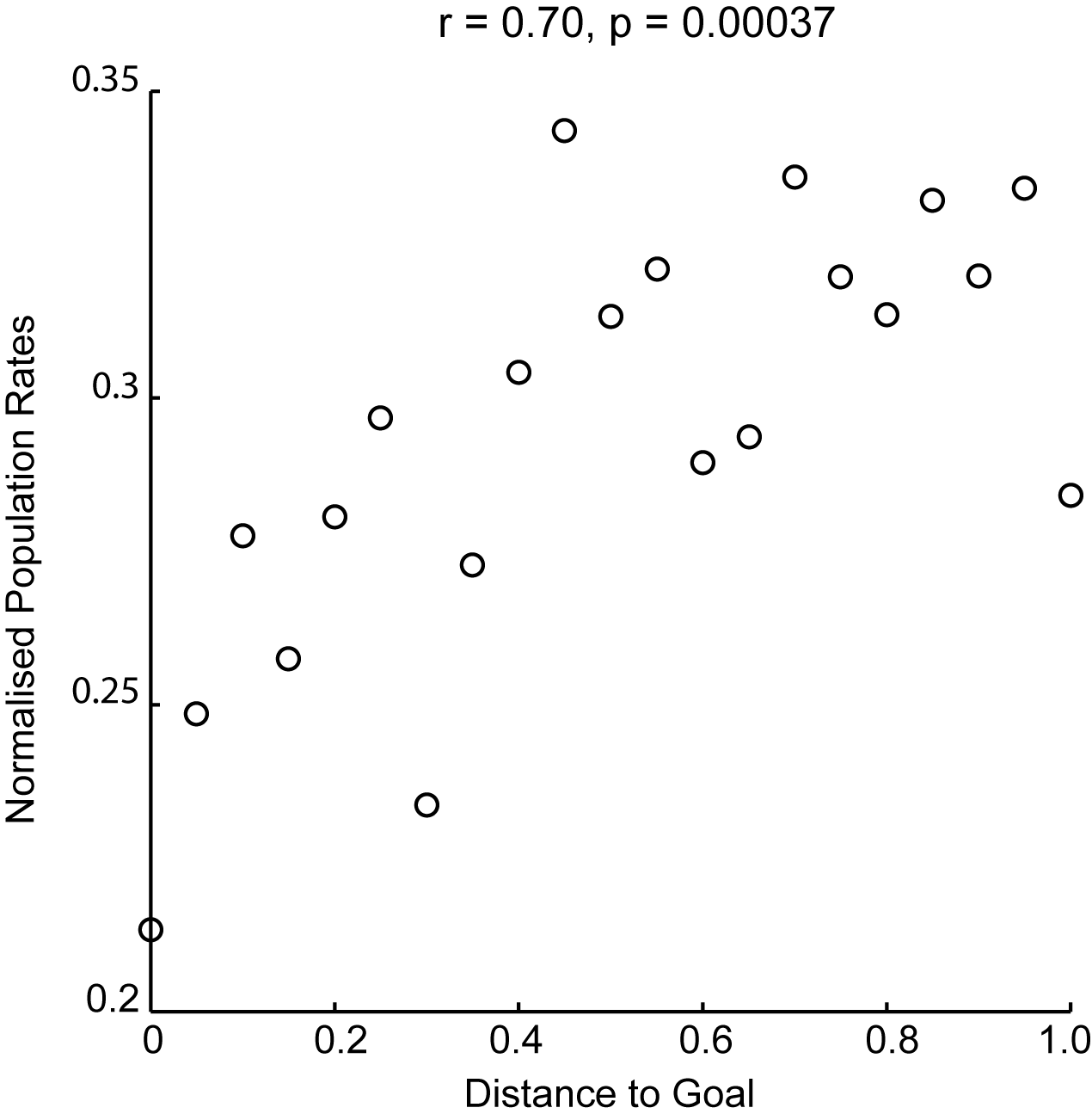

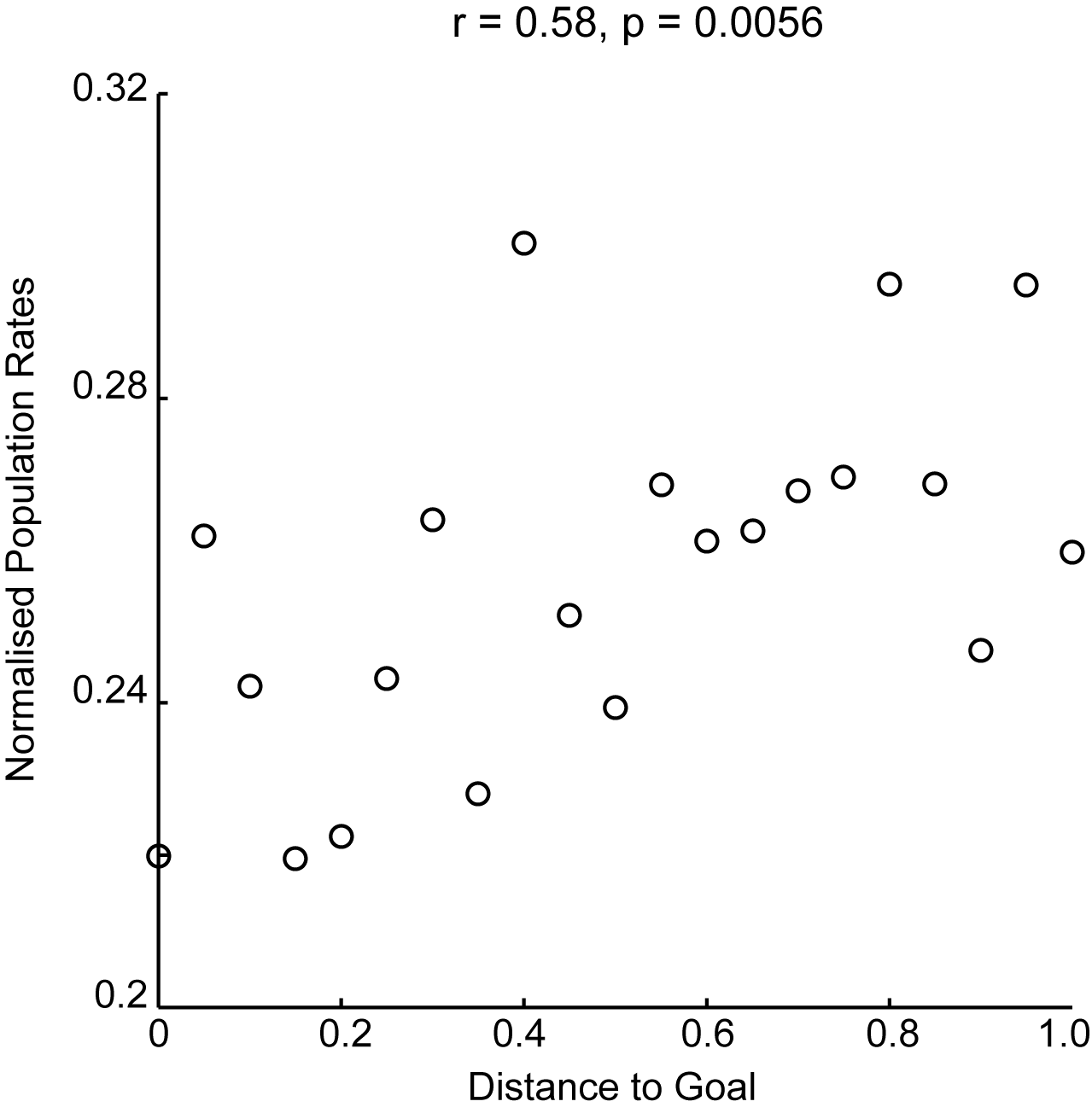


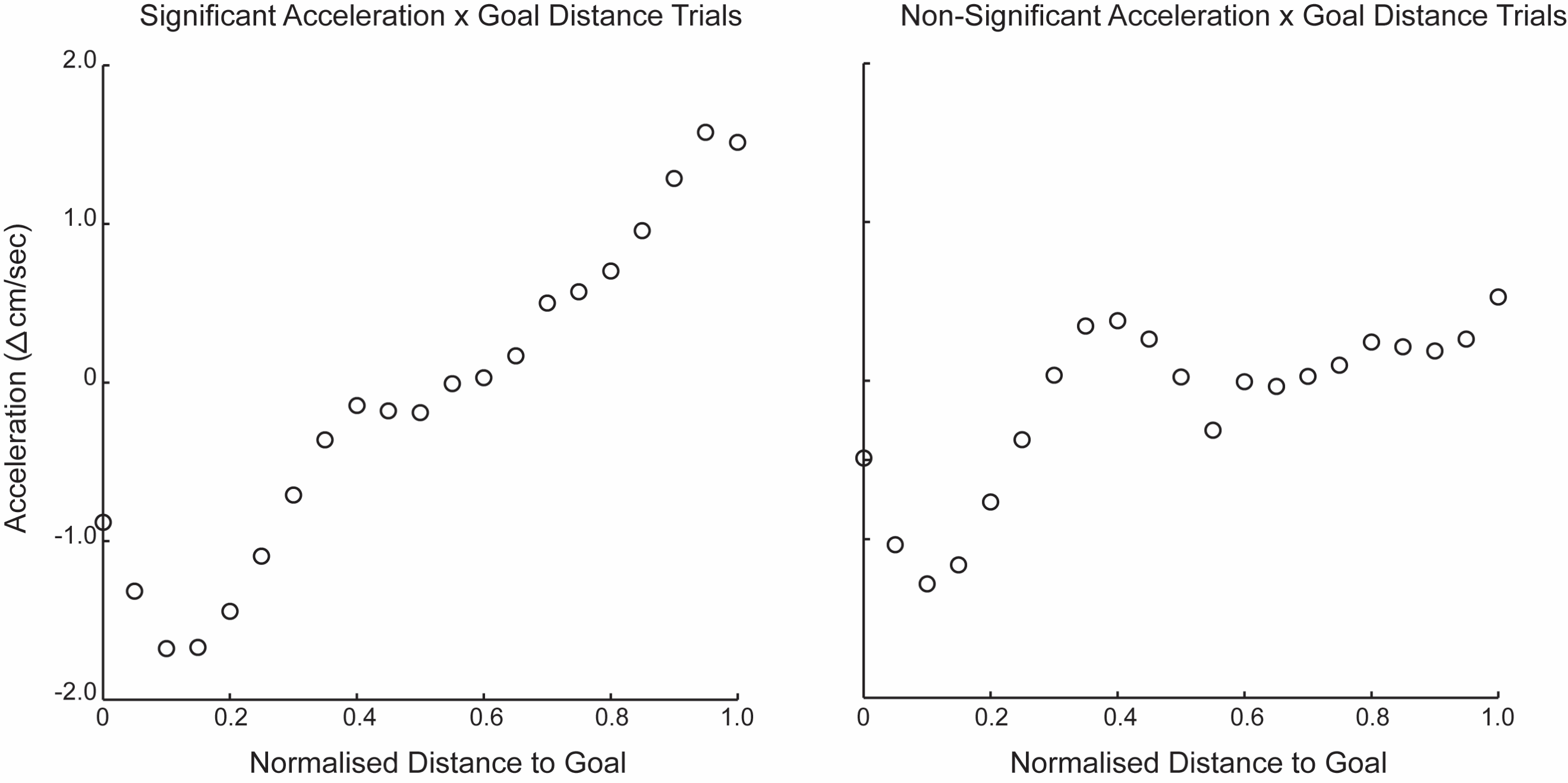


**Figure S2. The relationship between population activity and distance to goal when segregating trials on the basis of the correlation between acceleration and distance. Top Left)** Correlation between population rates and distance to goal for trials in which acceleration was significantly correlated with the distance to the goal. **Top Right)** Correlation between population rates and distance to goal for trials in which acceleration was not significantly correlated with the distance to the goal. The title shows the correlation between the two axes. **Bottom Left**) Acceleration plotted against normalised distance to goal for trials with a significant correlation between acceleration and distance to goal. **Bottom Right**) Acceleration plotted against normalised distance to goal for trials without a significant correlation between acceleration and distance to the goal.


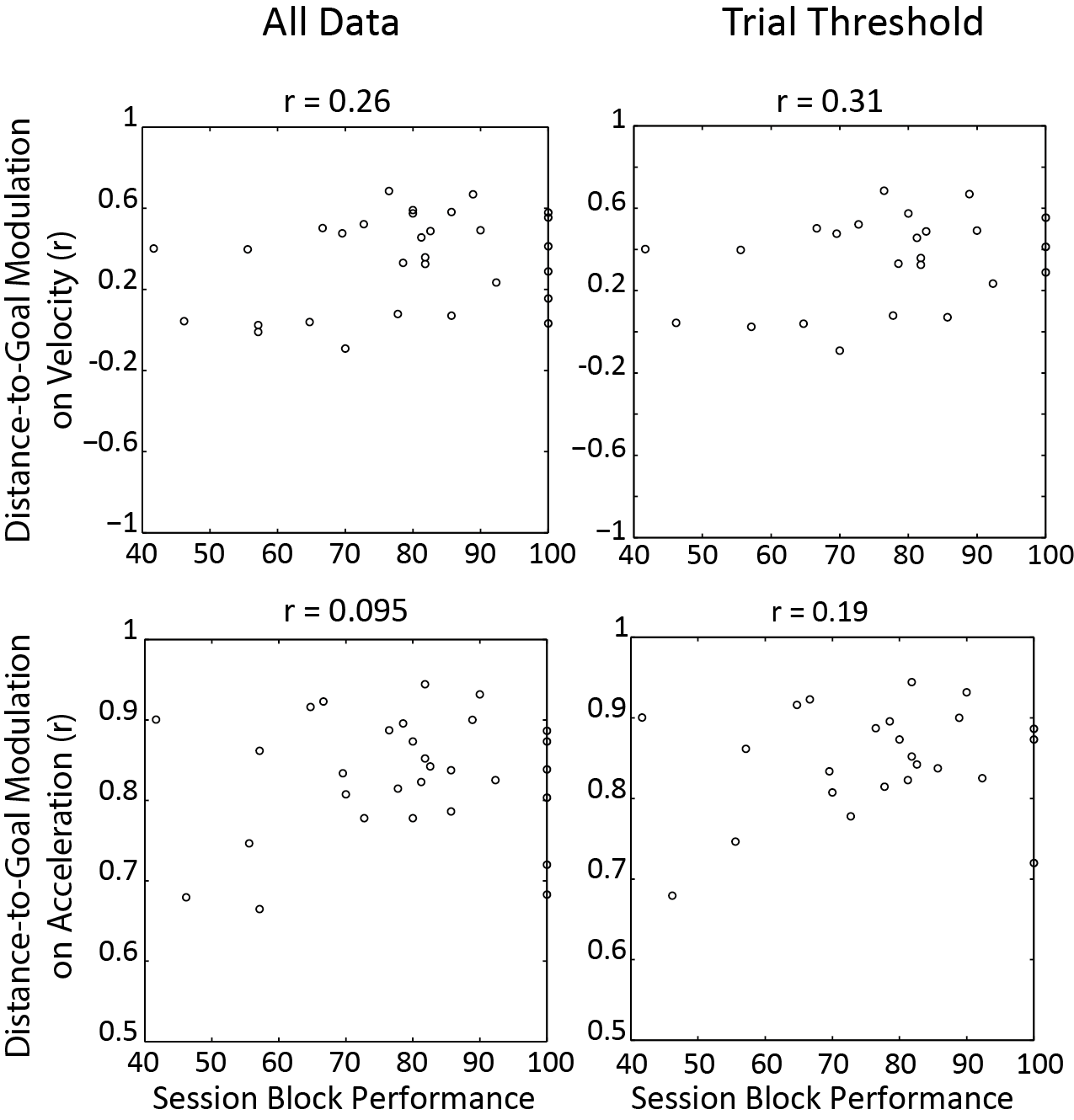


**Figure S3. The Influence of Navigational Performance over the Relationship between Running Speed/Acceleration and Distance to Goal. Top Left)** Session block correlation between velocity and distance to goal plotted against session block performance. **Top Right)** Same as Top Left but excluding session blocks with eight or less trials. **Bottom Left)** Same as Top Left but for the session block correlations between acceleration and distance to goal. **Bottom Right)** Same as Bottom Left but excluding session blocks with eight or less trials. X-axis shows session block performance, y-axis correlation between running speed/acceleration and distance to goal. The title shows the correlation between the two axes.


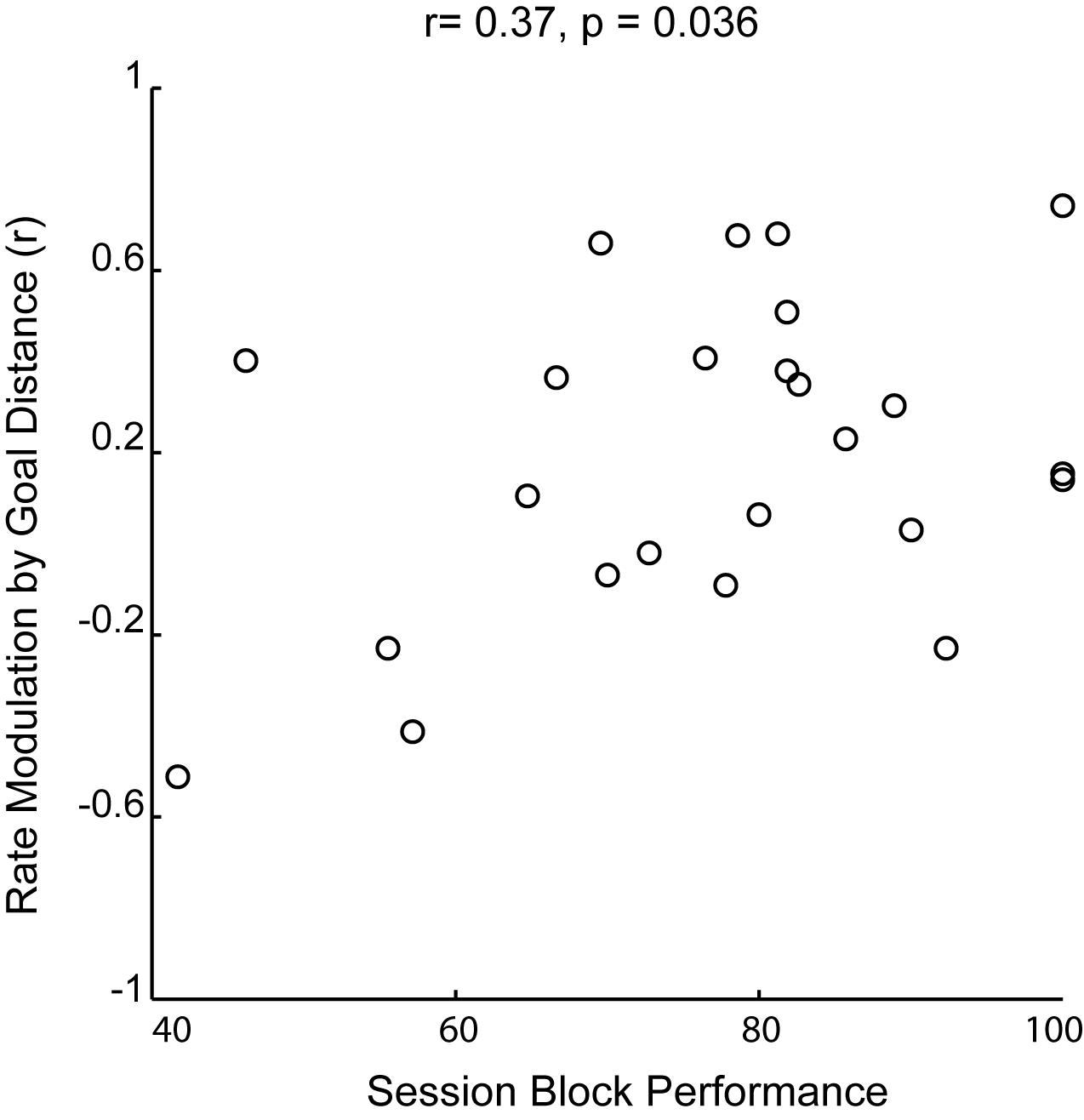


**Figure S4. The relationship between firing rate modulation by goal distance and performance for trials where acceleration was not significantly correlated with the distance to the goal.**
